# Supplementary material for: The Systems Biology Research Tool: evolvable open-source software
Source: BMC Syst Biol. 2008 Jun 29;2:55. doi: 10.1186/1752-0509-2-55 (PMC2446383; doi:10.1186/1752-0509-2-55)
Supplement: Additional file 1 — SBRT Archive. An archive of the current version of the Systems Biology Research Tool. [file 1752-0509-2-55-S1.zip › sbrt-1.4.0/doc/developers_guide/api/sbrt/kernel/math/algebra/SparseDoubleMatrix.html]

SparseDoubleMatrix


|  |  |  |
| --- | --- | --- |
| **Overview** \| **Package** \| **Class** \| **Use** \| **Tree** \| **Deprecated** \| **Index** \| **Help** | |  |
| **PREV CLASS**   **NEXT CLASS** | **FRAMES**    **NO FRAMES**     **All Classes** |  |
| SUMMARY: NESTED \| FIELD \| CONSTR \| METHOD | DETAIL: FIELD \| CONSTR \| METHOD |  |


---


## sbrt.kernel.math.algebra Interface SparseDoubleMatrix<R,C>

**Type Parameters:**: `R` - the row type.: `C` - the column type.

**All Superinterfaces:**: DoubleMatrix<R,C>, Matrix<R,C,java.lang.Double>

**All Known Subinterfaces:**: SmdMatrix<R,C>

**All Known Implementing Classes:**: AbstractSmdMatrix, HashMatrix, RowOrTreeMatrix, SparseDoubleMatrixWrap, TreeMatrix

---

``` public interface SparseDoubleMatrix<R,C> extends DoubleMatrix<R,C> ```

This interface is used to represent sparse matrices whose
elements are strictly double precision numbers.

**Author:**
:   This interface was written and documented by
    Jeremiah Wright while in the Wagner lab.

---

| **Method Summary** | |
| --- | --- |
| `java.util.Set<C>` | `getColIndNzEl(java.lang.Object rowIndex)`             Returns the set of column indices whose elements are nonzero in the row corresponding to the provided index. |
| `SparseDoubleMatrix<R,C>` | `getColumn(java.lang.Object columnIndex)`             Returns the column corresponding to the provided column index. |
| `SparseDoubleMatrix<R,C>` | `getRow(java.lang.Object rowIndex)`             Returns the row corresponding to the provided row index. |
| `java.util.Set<R>` | `getRowIndNzEl(java.lang.Object columnIndex)`             Returns the set of row indices whose elements are nonzero in the column corresponding to the provided index. |

**Methods inherited from interface sbrt.kernel.math.algebra.DoubleMatrix**

`getElement, toLinearCombs`

**Methods inherited from interface sbrt.kernel.math.algebra.Matrix**

`columns, equals, getColumnIndices, getRowIndices, hashCode, isColumnIndex, isEmpty, isRowIndex, rows, toString`

**Method Detail**

### getRowIndNzEl

```
java.util.Set<R> getRowIndNzEl(java.lang.Object columnIndex)
```

:   Returns the set of row indices whose elements are nonzero
    in the column corresponding to the provided index.

    :   **Parameters:**: `columnIndex` - the column index. **Returns:**: the set of row indices whose elements are nonzero in the column corresponding to the provided index.

---


### getColIndNzEl

```
java.util.Set<C> getColIndNzEl(java.lang.Object rowIndex)
```

:   Returns the set of column indices whose elements are nonzero
    in the row corresponding to the provided index.

    :   **Parameters:**: `rowIndex` - the row index. **Returns:**: the set of column indices whose elements are nonzero in the row corresponding to the provided index.

---


### getRow

```
SparseDoubleMatrix<R,C> getRow(java.lang.Object rowIndex)
```

:   Returns the row corresponding to the provided row index.
    A row is itself a 1 x n matrix, where n is the number of
    columns in this matrix.

    :   **Specified by:**: `getRow` in interface `DoubleMatrix<R,C>` **Specified by:**: `getRow` in interface `Matrix<R,C,java.lang.Double>`
    :   **Parameters:**: `rowIndex` - the index of the row to be returned. **Returns:**: the row corresponding to the provided row index.

---


### getColumn

```
SparseDoubleMatrix<R,C> getColumn(java.lang.Object columnIndex)
```

:   Returns the column corresponding to the provided column index.
    A column is itself an m x 1 matrix, where m is the number of
    rows in this matrix.

    :   **Specified by:**: `getColumn` in interface `DoubleMatrix<R,C>` **Specified by:**: `getColumn` in interface `Matrix<R,C,java.lang.Double>`
    :   **Parameters:**: `columnIndex` - the index of the column to be returned. **Returns:**: the column corresponding to the provided column index.


---


|  |  |  |
| --- | --- | --- |
| **Overview** \| **Package** \| **Class** \| **Use** \| **Tree** \| **Deprecated** \| **Index** \| **Help** | |  |
| **PREV CLASS**   **NEXT CLASS** | **FRAMES**    **NO FRAMES**     **All Classes** |  |
| SUMMARY: NESTED \| FIELD \| CONSTR \| METHOD | DETAIL: FIELD \| CONSTR \| METHOD |  |


---
